# Fluorescence Signal Enhancement in Antibody Microarrays Using Lightguiding Nanowires

**DOI:** 10.3390/nano11010227

**Published:** 2021-01-16

**Authors:** Damiano Verardo, Leena Liljedahl, Corinna Richter, Björn Agnarsson, Ulrika Axelsson, Christelle N. Prinz, Fredrik Höök, Carl A. K. Borrebaeck, Heiner Linke

**Affiliations:** 1 NanoLund, Lund University, Box 118, 22100 Lund, Sweden; damiano.verardo@gmail.com (D.V.); christelle.prinz@ftf.lth.se (C.N.P.); fredrik.hook@chalmers.se (F.H.); 2 Solid State Physics, Lund University, Box 118, 22100 Lund, Sweden; 3 AlignedBio AB, Medicon Village, Scheeletorget 1, 223 63 Lund, Sweden; 4 CREATE Health Translational Cancer Center, Department of Immunotechnology, Lund University, Medicon Village Bldg 406, 223 63 Lund, Sweden; liljedahl@gmail.com (L.L.); corinna.richter@immun.lth.se (C.R.); ulrika.axelsson@immun.lth.se (U.A.); carl.borrebaeck@immun.lth.se (C.A.K.B.); 5 Department of Physics, Chalmers University of Technology, 41296 Gothenburg, Sweden; dr.bjorn.agnarsson@gmail.com

**Keywords:** antibody microarray, nanowire sensors, biomarker discovery

## Abstract

Fluorescence-based detection assays play an essential role in the life sciences and medicine. To offer better detection sensitivity and lower limits of detection (LOD), there is a growing need for novel platforms with an improved readout capacity. In this context, substrates containing semiconductor nanowires may offer significant advantages, due to their proven light-emission enhancing, waveguiding properties, and increased surface area. To demonstrate and evaluate the potential of such nanowires in the context of diagnostic assays, we have in this work adopted a well-established single-chain fragment antibody-based assay, based on a protocol previously designed for biomarker detection using planar microarrays, to freestanding, SiO_2_-coated gallium phosphide nanowires. The assay was used for the detection of protein biomarkers in highly complex human serum at high dilution. The signal quality was quantified and compared with results obtained on conventional flat silicon and plastic substrates used in the established microarray applications. Our results show that using the nanowire-sensor platform in combination with conventional readout methods, improves the signal intensity, contrast, and signal-to-noise by more than one order of magnitude compared to flat surfaces. The results confirm the potential of lightguiding nanowires for signal enhancement and their capacity to improve the LOD of standard diagnostic assays.

## 1. Introduction

Due to their versatility and practical applicability, fluorescence-based readout assays play an essential role in life sciences and medicine [1], with applications spanning from live-cell imaging [2] and protein interaction kinetics and folding [3] to in vitro and in vivo medical diagnostics [4]. Despite their ubiquity, constant efforts are made to improve both their sensitivity and specificity. For instance, in the context of diagnostics [5], lowering the limit of detection (LOD) is central to achieving reliable identification of low-abundance biomarkers, which is a prerequisite for early diagnosis [6]. Efforts towards this aim include the development of high-sensitivity photodetectors [7], high-quantum yield and photostable fluorescent probes (both organic [8,9] and inorganic [10]), and more recently, efficient means to interface high-affinity biomolecular interaction partners with nanostructure-based fluorescence enhancement platforms.

The nanomaterials so far predominantly explored for the latter purpose are primarily metallic nanoparticles, providing plasmonic-induced fluorescent enhancement [11,12]. In the context of optical biosensor applications, semiconductor nanowires have been mostly explored on the basis of their higher surface area compared with planar substrates, but they have recently gained increased attention also due to their highly advantageous optical properties [13,14,15]. Specifically, nanowires have been shown to collect the fluorescence emission of a large number of surface-bound fluorophores and re-emit it at their tip, not unlike an optical fiber [16], thereby greatly enhancing the overall intensity of the emission and even enabling single-molecule detection without advanced optics [17]. In addition to the effect of the increased surface area offered by the nanowires, which increases the total fluorescence signal within the field-of-view, the signal is furthermore enhanced due to a combination of advantageous physical effects: (i) the relatively high refractive index of semiconductor nanowires enables them to support waveguide modes at small diameters, collecting and guiding the emission from fluorophores placed in immediate proximity to the nanowire surface [16,18]; (ii) it has been suggested that the coupling of excitation light in waveguide modes can increase the intensity of the local electromagnetic field close to the nanowire’s surface, enhancing the excitation of fluorophores placed in their close proximity [19,20]; and (iii), the emission of light from the nanowire tip can be highly directional, thereby increasing the light collection efficiency of the readout system.

Several studies, employing different semiconductor materials (e.g., ZnO [21], Si [22], GaP [14], InAs [23], GaAs [19]), have addressed the potential of nanowires to enhance fluorescent signals. Among them, some investigated the physical interactions between nanowires and fluorescent dyes suitable for biomolecular labeling [15,16,18,20], for example showing how guiding efficiency is strongly dependent on nanowire material, diameter, and light wavelength, with different diameters maximizing efficiency for a certain wavelength range, allowing optimization for selected fluorophores [16]. Others explored their use for investigating biological systems, such as molecular motors [14], supported lipid bilayers [17], and live cells [19]. Patterned arrays of spatially ordered ZnO nanowires have been used for protein microarray applications, demonstrating detection limits in the fM regime, even beating state-of-the-art ELISA platforms when applied both on human samples and serum spiked with carefully controlled biomarker concentrations [20,24]. Although the exact magnitude of the fluorescence enhancement provided by nanowires has been subject to intense evaluations [20], quantitative measurements of the enhancement provided by nanowires have been so far scarce, in terms of immunoassays tested, and focused on ZnO nanowires [20,24]. Compared to ZnO, the GaP NWs used in this study have a considerably higher refractive index, allowing them to support waveguide modes at smaller diameters and guide light more efficiently than their ZnO counterparts. Moreover, in contrast to solution-processed ZnO nanowires, GaP wires can be synthesized using epitaxy, which allows for enhanced control over the nanowire size and shape, resulting in a homogeneous nanowire population with geometries precisely matched for optimal signal enhancement.

In many fluorescence-based detection assays, including antibody-based microarrays, a high signal intensity does not necessarily imply reliable data, because what matters is primarily the relative intensity of the signal compared to background. For this reason, contrast and signal-to-noise ratio are preferred quantities to use when evaluating how well a signal of interest can be resolved [25]. In this work, we therefore use these measures to assess the capacity of SiO_2_-coated GaP nanowires for the detection of biomarkers using a well-established immunoassay originally designed for flat surfaces [26] and proven capable of identifying unique biomarker signatures for cancer diagnostics [27,28]. Specifically, the nanowire platform is compared with flat, SiO_2_-coated silicon substrates, and to MaxiSorp plastic slides, the latter being the standard platform for this particular single chain fragment variable (scFv) antibody-based microarray assay [26,29]. ScFv can easily be produced in bacterial culture and we have earlier shown their high suitability for both the generation of libraries and the use in high-throughput multiplexed applications [26,30], allowing to assess how nanowires could impact a state-of-the-art system. Moreover, the fact that ScFv are well adsorbed on negatively charged surfaces (such as MaxiSorp or SiO_2_) makes the testing of silica-coated nanowire samples possible without any additional surface functionalization. The performance of the three types of surfaces named above is evaluated using five different scFv antibodies directed against different biomarkers, the expression of which being correlated with several different diseases, for example lung and pancreas cancer (CEACAM-1) [31,32], asthma (IL-5) [33], and heart disease (MCP-1) [34]. For these biomarkers, we analyze the total fluorescent signal intensity, the Weber contrast, and the signal to noise ratio (SNR) and find that all three parameters are more than an order of magnitude larger on nanowire substrates compared to flat substrates. Moreover, at low serum concentrations, where no signal could be detected on flat substrates, a quantifiable signal could still be obtained on nanowires. Taken together, our results show that lightguiding nanowire substrates have a significant potential as a platform for ultrasensitive, fluorescence-based biomarker detection when low detection limits are needed.

## 2. Materials and Methods

### 2.1. GaP Nanowires Synthesis

**Figure 1:**
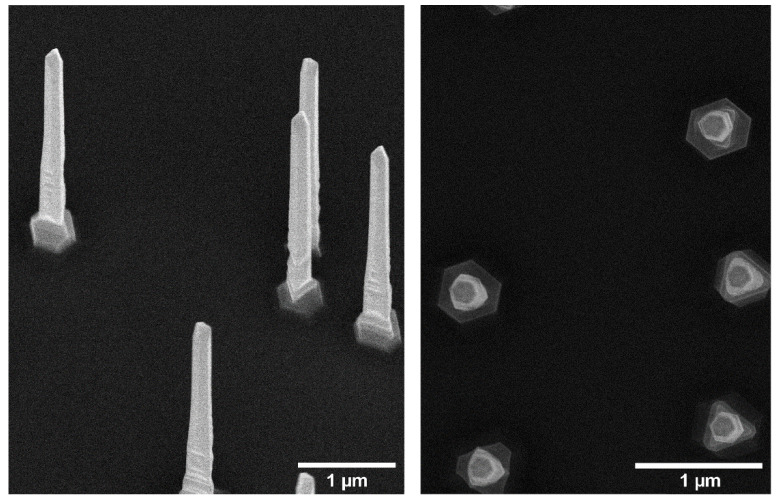
Scanning electron microscopy images of the GaP nanowires used in this study, taken at 30° and 0° stage inclination (perpendicular view).

GaP nanowires were grown on (111)B GaP substrates using metalorganic vapor phase epitaxy (MOVPE) from 50 nm Au seed nanoparticles randomly deposited on the substrate at a density of approximately 0.35 µm^−2^ [35]. At this density, the average distance between nanowires is ~3 μm, sufficiently larger than one wavelength (~650 nm) to avoid waveguide mode superposition between nanowires. Two batches of nanowire substrates were used in this study, grown (i) axially to a length of 4.1 ± 0.1 µm and then radially to a diameter of 160 ± 7 nm and (ii) to a length of 3.7 ± 0.1 µm and a diameter of 160 ± 7 nm, respectively (Figure 1). The specific diameter allows for optimal in-coupling of light emitted by fluorophores in the red spectral range (such as for the dye Alexa647 used in the current experiments), due to the presence of partially localized waveguide modes. The small variations in diameter between nanowires in the same batch are not expected to significantly impact their waveguiding properties, in particular considering the line width of the fluorophores [16]. Nanowire samples and flat Si substrates were coated with ~10 nm of silicon oxide (SiO_2_) using atomic layer deposition (ALD).

### 2.2. Biotinylation of Serum Samples

Biotinylation of serum was performed on ice or at 4 °C according to standard procedure. Briefly, serum samples were centrifuged at 16,000× *g* and the resulting cleared serum then diluted in PBS (D-PBS-Sterile *w*/*o* Mg, Ca, GE Healthcare Life Sciences, Marlborough, MA, USA) to 1:5 before the addition of biotin (EZ-LinkTM NHS-PEG4-Biotin, Thermo Fisher Scientific, Waltham, MA, USA) to a final concentration of 1.13 mM. The reaction was terminated after 2 h by adding Tris-HCl pH 8.0 (Thermo Fisher Scientific, Waltham, MA, USA) to a final concentration of 181 mM. For each biotinylation plate, three replicates of a reference serum sample (ERM-DA470k/IFCC, JRC, Geel, Belgium) were included as the process control. Biotinylated samples were pooled, aliquoted, and stored at −80 °C.

### 2.3. scFv Production

Human recombinant single-chain Fv antibodies were purified from *E. coli,* using His MultiTrap FF 96 well plates (GE Healthcare Life Sciences, Marlborough, MA, USA), according to the manufacturer’s instructions. Buffer exchange to PBS (GE Healthcare Life Sciences) was performed using ZebaTM 96-well desalt spin plates (Thermo Fisher Scientific, Waltham, MA, USA). Sodium azide (GBiosciences, Saint Louis, MO, USA) was added to the purified scFv in PBS to a final concentration of 0.06%. The purity of the scFvs was evaluated by SDS-PAGE, using 8–16% Criterion™ TGX Stain-Free™ Protein Gel (BioRad, Hercules, CA, USA). Concentrations were measured in a SPECTROstar Omega microplate reader at 280 nm and analyzed with the included MARS software (BMG Labtech, Ortenberg, Germany). PC013 and PC070 were produced at the Department of Immunotechnology, Lund University, Sweden, and RD003, RD004, and RD017 at Immunovia AB, Lund, Sweden.

### 2.4. Sample Functionalization

**Figure 2:**
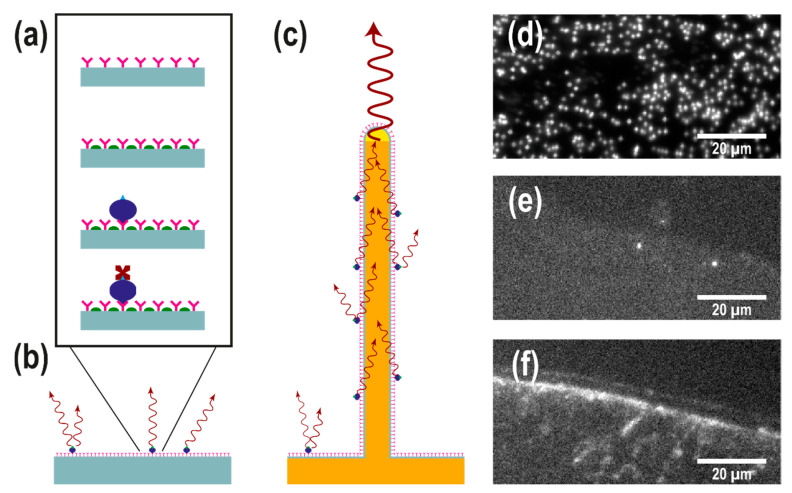
(**a**) Schematic representation of the single chain fragment variable (scFv) assay used in this study. ScFv antibodies (pink) are physisorbed on a surface. A blocking agent (green) is subsequently used to minimize unspecific binding of biotinylated serum proteins (blue), which are targeted by fluorescently labelled streptavidin (dark red). (**b**,**c**) Schematic representation of the assay on a flat substrate (**b**) and a light-guiding nanowire substrate (**c**): the emission of fluorophores on the nanowire surface excites the supported waveguide modes to be reemitted at the tip, leading to an enhanced signal intensity at the tip of the nanowires. (**d**–**f**) Top-view epifluorescence images (displayed in the same pixel window) of (**d**) a GaP nanowire substrate, (**e**) a silicon substrate and (**f**) a MaxiSorp black polymer substrate, spotted with antibodies and exposed biotinylated serum concentration diluted to 0.4% (the highest concentration used in this study). Individual nanowires are visible in (**d**) as bright spots. The edge of the scFv spot is visible in (**e**,**f**), showing the difference in the signal between specific an unspecific binding to the antibodies.

The assay used in this study was adapted from an assay developed for scFv microarrays [29] on black polymer MaxiSorp slides (NUNC A/S) [26]. The same assay was run on three different substrates: (i) SiO_2_-coated nanowire substrates, (ii) SiO_2_-coated, flat silicon substrates, and (iii) flat MaxiSorp slides, henceforth referred to as nanowire, silicon, and MaxiSorp substrates, respectively. Prior to scFv spotting, the nanowire and silicon substrates were ozone-preened for 40 min in order to clean and activate the surface, glued to a MaxiSorp slide, and placed in a 12-well slide holder. Spotting of antibodies was done by manually depositing 1.5 µL of scFv solution on each type of substrate. This resulted in the silicon and MaxiSorp substrates having only part of their surface exposed to the scFv solution. In contrast, the smaller and more hydrophilic nanowire substrates were entirely covered by the scFv solution. All substrates were then incubated at room temperature in a moist environment for about 20 h in stagnant conditions to facilitate scFv adsorption. After incubation, the substrates were rinsed thoroughly with 0.05% Tween 20 solution in PBS to remove the excess antibodies, then incubated on an orbital shaker for one hour with a blocking solution (1 w% fat-free milk powder, 1% Tween solution in PBS) to passivate the surface. The substrates were then rinsed thoroughly with 0.05% Tween solution in PBS and incubated for two hours in biotinylated serum diluted in blocking solution. The substrates were rinsed four times with 0.05% Tween solution in PBS and incubated on an orbital shaker with 10 µM Streptavidin-Alexa 647 in blocking solution for one hour, then rinsed with MilliQ water and dried with nitrogen (see Figure 2a–c for a schematics of the assay). All incubation steps were performed at room temperature while shielding the samples from light.

### 2.5. Sample Imaging

All samples were imaged within a few hours from preparation in epifluorescence using a custom-built microscope, a 40×, 0.85 NA air objective (Nikon, Tokyo, Japan), a Prime95B scientific CMOS camera (Photometrics, Tucson, AZ, USA), and a high-power LED source (Thorlabs, Newton, NJ, USA) for illumination (emission peak at 625 nm). The lamp power was kept at 15% (~15 mW) to limit bleaching. The camera exposure time was set to 500 ms (See Figure 2d–f for raw images of the signal collected from each substrate). A total of five different scFv were used in this study and duplicates were made for each scFv and for each substrate. The scFv target and spotting concentrations are reported in Table 1. Henceforth, each scFv type will be referred to by its tag number.

In the standard protocol used for these scFv, biotinylated serum is further diluted to 2% in blocking solution. However, to better test the enhancement provided by nanowires, the standard solution was further diluted by factors of 1:5 (0.4%), 1:10 (0.2%), and 1:20 (0.1%), henceforth referred to as 0.4%, 0.2%, and 0.1%, respectively. Two control samples were prepared for each substrate type to estimate nonspecific binding: one by exposing scFv-spotted substrates to a solution containing no serum (0%), and one by exposing non-spotted surfaces to the highest serum concentration used in this study (0.4%).

**Figure 3:**
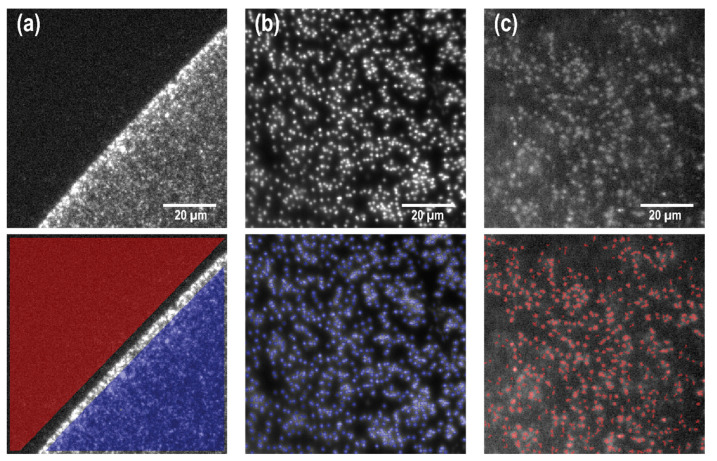
Image analysis for signal extraction on (**a**) MaxiSorp black polymer plastic spotted with antibodies and exposed to 0.4% serum concentration, (**b**) scFv-spotted nanowires exposed to 0.4% serum concentration, and (**c**) non-spotted nanowires exposed only to 0.4% serum concentration (control sample). Panel (**a**) is displayed in a different pixel window than panels (**b**,**c**) to better show the contrast between spotted and non-spotted areas in (**a**). On flat substrates, the signal and background intensity are extracted as the mean pixel value in the areas inside (blue) and outside (red) the scFv spot, respectively. On nanowire samples, *I_s_* is the mean pixel intensity on nanowire positions, (highlighted in blue in (**b**)). Similarly, *I_B_* is the mean pixel intensity on nanowire positions on the control substrate (red in (**c**)).

For silicon and MaxiSorp substrates, where the edge of the scFv spot could be identified (Figure 2d,e), the signal intensity *I_s_* and background intensity *I_B_* were extracted as the mean pixel value corresponding to regions inside and outside the scFv spot, respectively (Figure 3a).

However, due to the non-homogeneous nature of the nanowire substrates, the analysis on nanowires had to be performed in a slightly different manner. In the analysis, wires were defined as regions containing more than five bright pixels after the image had been processed with a Laplacian and threshold filters. The signal intensity, *I_s_*, for each wire was then calculated as the mean pixel value of the region defining the wire (Figure 3b). Because SiO_2_-coated nanowire substrates are very hydrophilic after ozone preening, and since the samples used in our study are relatively small (<5 mm^2^), the spotting of scFv fragments on a nanowire substrate inevitably resulted in the scFv fragment drop covering the entire surface of the sample, making it impossible to extract *I_B_* from a non-spotted area on the same substrate. Instead, the background intensity *I_B_* on nanowire substrates was designed as the mean pixel value at the positions of nanowires on substrates that were not exposed to scFv fragments but exposed to serum only (Figure 3c).

## 3. Results

The assay used in this study was originally developed for antibody microarrays [29] on black polymer MaxiSorp slides (NUNC A/S) [26], and it was run on three different substrates: (i) SiO_2_-coated nanowire substrates, (ii) SiO_2_-coated, flat silicon substrates and (ii) flat MaxiSorp slides (see Figure 2). A total of five different scFv (Table 1) were spotted manually on the different substrates followed by surface passivation, incubation in biotinylated serum, and incubation in Streptavidin-Alexa Fluor 647 (see Materials and Methods for detailed experimental protocol) before imaging by fluorescence microscopy.

**Figure 4:**
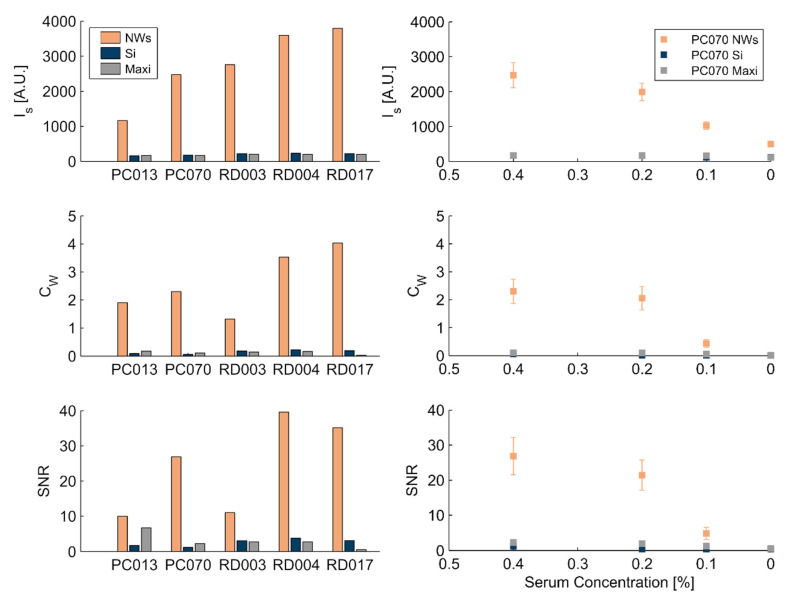
Left: extracted values of signal intensity *I_s_*, Weber contrast *C_W_*, and signal to noise ration (*SNR*) for the various scFvs on the three substrates tested with 0.4% serum concentration. All three parameters are consistently higher on the nanowire substrates. Right: example of the effect of serum concentration for the PC070 scFv. Decreasing the serum concentration results in a decrease in the signal, with 0.1% concentration approaching the limit for reliable signal on nanowires. Data for all tested scFvs are shown in the Figure S1. Comparison graph in logarithmic scale are shown in the Figure S2.

Signal intensity, Weber contrast, and signal-to-noise ratio were extracted from the acquired microscopy images of the three different substrate types after rinsing in water and drying in nitrogen. The Weber contrast *C*_W_ and the signal-to-noise (*SNR*) were evaluated as

(1) $$C_{W}=\frac{I_{S}-I_{B}}{I_{B}}$$

and

(2) $$SNR=\;\frac{I_{S}-I_{B}}{\sigma_{B}}$$

where *σ_B_* is the pixel-value standard deviation of the background. The Weber contrast *C_W_*, which is also commonly referred to as the signal-to-background ratio in the literature, is a measure of the relative intensity of the signal compared to the background. The signal-to-noise ratio (sometimes referred as the signal-to-standard-deviation ratio, or *SSR*) is instead a measurement of how well the signal stands out over the noise fluctuations. *I*_S_, *C*_W_, and *SNR* for the three substrates and the five scFv types after exposure to 0.4% serum concentration are plotted in Figure 4 (left).

For each scFv and serum concentration, the flat silicon and MaxiSorp substrates exhibit almost undetectable fluorescent signal intensities. In contrast, *I*_S_ on nanowires is higher by a factor 10–20 compared to flat surfaces, in agreement with previous findings obtained on ZnO nanowires [20]. Similarly, the Weber contrast and *SNR* are higher on nanowires (*C*_W_ ≈ 2–3; *SNR* > 10) compared to flat surfaces (*C*_W_ ≈ 0.1–0.2; *SNR* ≈ 1–2). For comparison, in the case of DNA microarrays, a commonly applied positive detection threshold is set to *C*_W_ ≈ 0.5 [43], and *SNR* ≈ 3 [44].

Reduction of the biomarker concentrations by decreasing the serum concentration results in a lowered signal intensity, Weber contrast, and SNR for all antibodies. For the most diluted samples tested (0.1%), it was in fact not possible to effectively measure the signal intensity on silicon and MaxiSorp substrates for some antibodies, since it was too close to that of the background to discern the edges of the scFv droplet (see Figure 3e,f). In contrast, for the same serum concentration, nanowires consistently exhibited higher signals than the background (Figure 4, right column).

## 4. Discussion

We used lightguiding, free standing GaP nanowires in a scFv antibody-based immunoassay and tested five different antibodies for the detection of biomarkers in human serum. We compared the signal intensity, signal contrast, and *SNR* obtained on nanowires compared to those on commonly used flat substrates. For all antibodies, the increase in signal intensity, contrast, and *SNR* is more than one order of magnitude larger on the nanowires compared to flat silicon and MaxiSorp substrates, proving that GaP nanowires can be used effectively for signal enhancement in immunoassays. Control experiments (i.e., spotted samples not exposed to serum and not-spotted samples exposed to serum) showed a consistently lower fluorescent signal compared to the respective experiments, verifying that unspecific binding to the surfaces played an insignificant role. Moreover, signal intensity of control experiments showed limited variations between experiments. The observed variation in *SNR* and *C_W_*, between different antibodies, can be attributed to differences in the adsorption of different antibodies and in the respective biomarker concentration. In addition, the process of manually spotting the samples also could contribute to signal variations, since manual spotting is inherently more prone to deviations than, for example, inkjet printing, which is commonly used to produce protein microarrays. Sample drying could have an effect as well, as the nanostructured surfaces cannot be blow-dried at high pressure.

It is worth noting that the assay used in this study is routinely used for antibody microarrays on MaxiSorp slides and was implemented on nanowires with minimal deviation from the routine protocol. The majority of the nanowires go through the process of repeated rinsing and drying steps with little or no damage. This shows that nanowire substrates are a sturdy platform, allowing for an easy implementation of pre-existing assays. In addition, the use of ALD enables the coating of nanowires with different materials, depending on surface chemistry needs. Altogether, our results indicate that using semiconductor nanowire substrates instead of standard flat substrates with fluorescence detection assays will improve the limit of detection, making them valuable for the detection of low abundance molecules in biological fluids. The results presented here are general to other types of nanowires made of high refractive index materials, as the lightguiding properties of nanowires depend directly on the refractive index difference between the nanowire and surrounding medium.

## 5. Conclusions

It is clear from the results presented in this study that the high surface-to-footprint ratio of the nanowires, combined with their lightguiding properties, could be used to greatly increase the detection limit of immunoassays or, alternatively, to decrease the amount of biological material required. Moreover, the increased signal quality on nanowires at very low serum concentrations opens up possibilities for developing a platform able to reliably detect very low abundance biomarkers in complex proteomes, important in, for example, the context of early-stage cancer detection [27,45] or miniaturization of diagnostic tools [46]. Although the aim of this work was not to quantify the limit of detection in terms of target concentration, which requires biomarker-free serum spiked with pure biomarker, it is worthwhile noting that while the serum dilution for the standard protocol developed for antibody microarray on the MaxiSorp Plastic is 2%, a fluorescent signal appreciably larger than the background could be detected on nanowires exposed to serum 20 times less concentrated (0.1%). For example, the typical concentration of MCP-1 (targeted by PC070) is on the order of 100 pg/mL, while that of IL-5 (targeted by PC070) is on the order of 10 pg/mL. A 20-fold increase in sensitivity raises the prospect of a limit of detection below 1 pg/mL (i.e., ~10–100 fM), which is comparable with state-of-the-art immunoassays [47]. Further work is necessary to precisely estimate the gain in LOD using a nanowire substrate, but these results prove how nanowires can substantially improve detection in a realistic assay (i.e., using human serum with native biomarker). In light of the fact that high-quality nanowire platforms are now becoming available at low cost, this makes this approach highly attractive in the intense search for simple biosensor formats offering reliable detection of low-abundance biomarkers.

## Figures and Tables

**Table 1:** Target and spotting concentration of single chain fragment variable (scFv), with typical biomarker concentrations in patients.

ScFv	Target Biomarker	Spotting scFv Conc. (mg/mL)	Typical biomarker Concentration (pg/mL)
PC013	Interleukin-5 (IL-5)	0.111	~10 (asthmatic) [33] ~1 (healthy) [36]
PC070	Monocyte chemoattractant protein (MCP-1)	0.139	~5 × 10^2^ (diabetic) [37] ~4 × 10^2^ (ischemic stroke) [34] ~10^2^ (healthy) [38]
RD003	Carcinoembryonic antigen-related cell adhesion molecule 1 (CEACAM-1)	0.300	~ 5 × 10^5^ (lung cancer) [31] ~1.5 × 10^5^ (melanoma) [39] ~6 × 10^5^ (healthy) [40]
RD004	Carcinoembryonic antigen-related cell adhesion molecule 1 (CEACAM-1)	0.300	
RD017	Cytotoxic T-lymphocyte-associated protein 4 (CTLA-4)	0.230	Large dynamic range reported: ~10^2^ (cancer patients) [41] ~2 × 10^3^ (primary proliferative glomerulonephritides) [42]

## Data Availability

The data presented in this study are available in Supplementary Information.

## Supplementary Materials

Supporting Information contains results for all the tested serum concentrations for all the antibodies tested and Figure 4 displayed in logarithmic scale. The following are available online at https://www.mdpi.com/2079-4991/11/1/227/s1, Figure S1: Data for all tested scFvs, Figure S2: Figure 4 plotted in logarithmic scale.
