# Supplementary material for: Fluorescence Signal Enhancement in Antibody Microarrays Using Lightguiding Nanowires
Source: Nanomaterials (Basel). 2021 Jan 16;11(1):227. doi: 10.3390/nano11010227 (PMC7829981; doi:10.3390/nano11010227)
Supplement: Supplementary file 1 [file nanomaterials-11-00227-s001.pdf]

nanomaterials

MDPI

Supplementary Information

Fluorescence Signal Enhancement in Antibody Microarrays Using Lightguiding Nanowires

Damiano Verardo[1,2,3], Leena Liljedahl[4], Corinna Richter[4], Björn Agnarsson[5], Ulrika Axelsson[4], Christelle N. Prinz[1,2], Fredrik Höök[1,5], Carl A.K. Borrebaeck[4], Heiner Linke[1,2] *

1. NanoLund, Lund University, Box 118, 22100 Lund, Sweden
2. Solid State Physics, Lund University, Box 118, 22100 Lund, Sweden,
3. AligND Systems, Medicon Village, Scheeletorget 1, 223 63 Lund, Sweden,
4. CREATE Health Cancer Center, Department of Immunotechnology, Lund University, Medicon Village Bldg 406, 223 63 Lund, Sweden,

* Correspondence: heiner.linke@ftf.lth.se

1. Data for all tested scFvs

The following graphs show extracted values of signal intensity Is, Weber contrast CW and signal to noise ration SNR for the various scFvs on the three substrates tested for all the tested serum concentrations. Only PC070 is reproduced in the main text as example in Fig 4.

In all the following graphs nanowires are indicated as NWs (organge), flat silicon wafer as Si (blue) and MaxiSorp plastic as Maxi (gray).

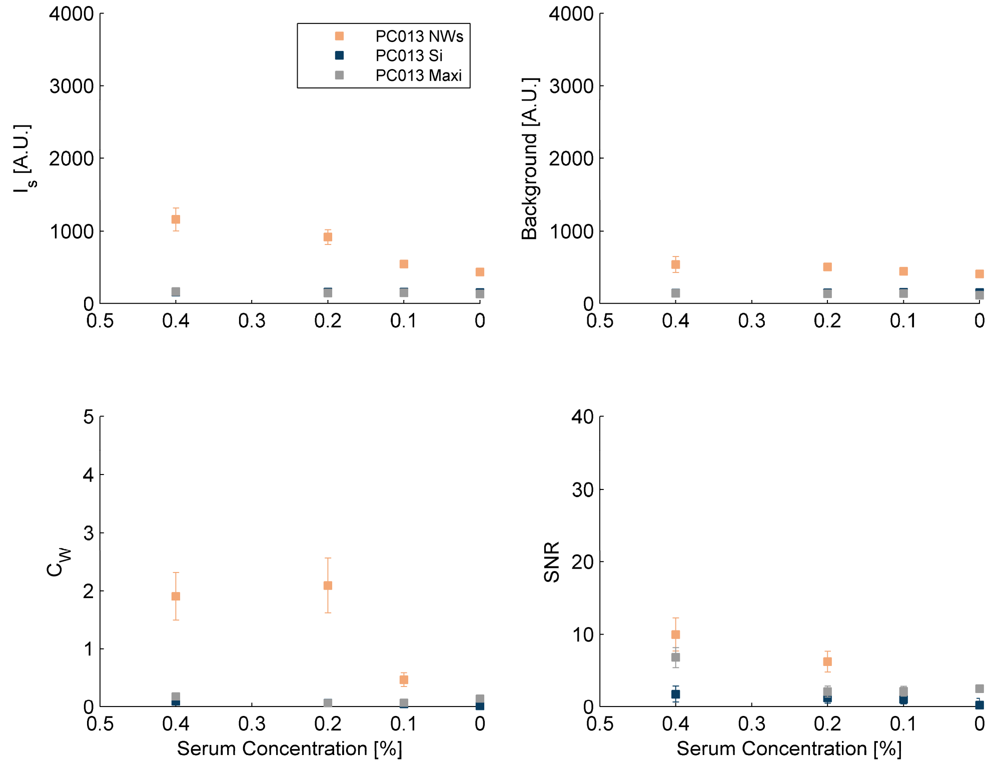
PC013 NWs
PC013 Si
PC013 Maxi
I_s [A.U.]
Background [A.U.]
C_W
SNR
Serum Concentration [%]
Serum Concentration [%]

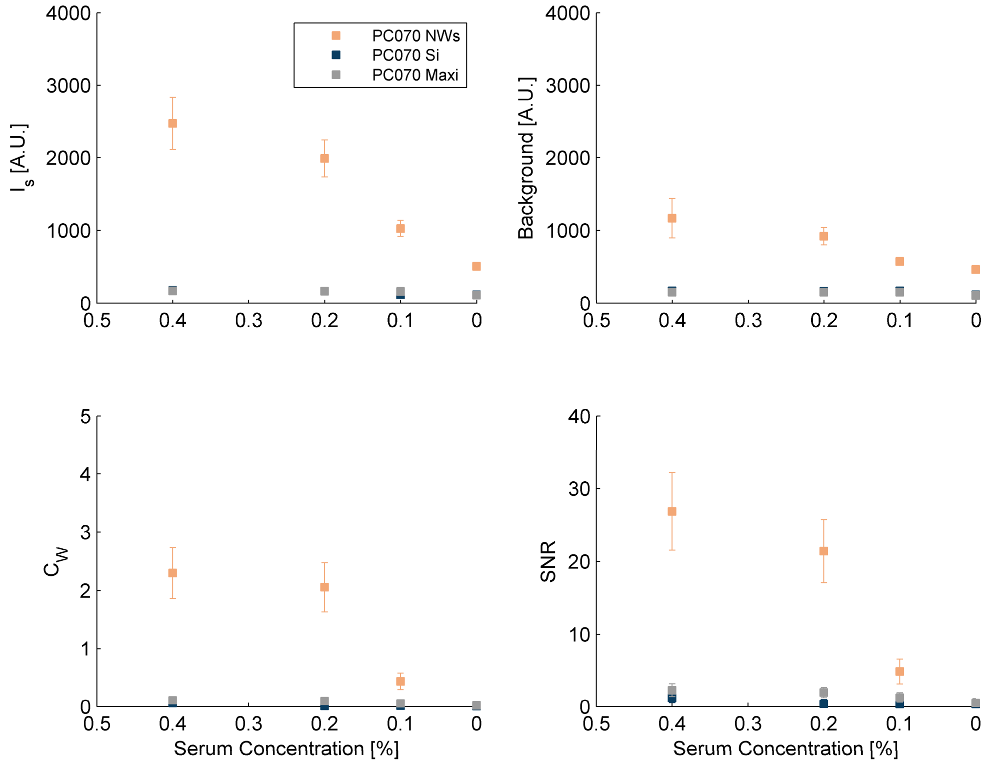
PC070 NWs
PC070 Si
PC070 Maxi
I_s [A.U.]
Background [A.U.]
C_W
SNR
Serum Concentration [%]
Serum Concentration [%]

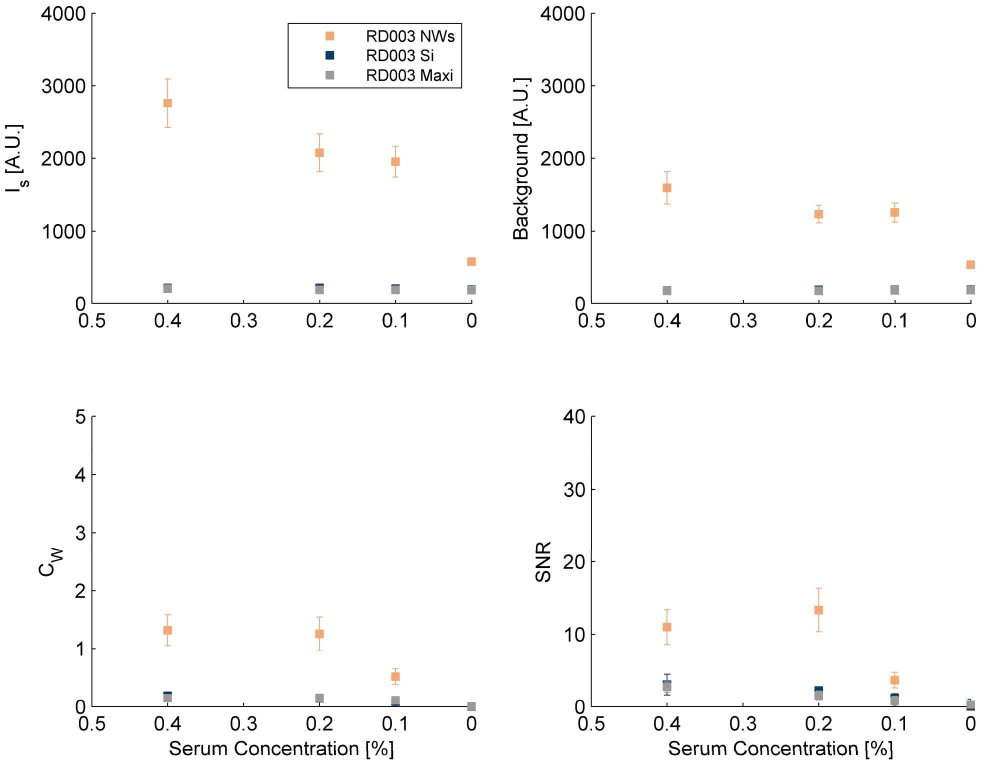
RD003 NWs
RD003 Si
RD003 Maxi
I_s [A.U.]
Background [A.U.]
C_W
SNR
Serum Concentration [%]

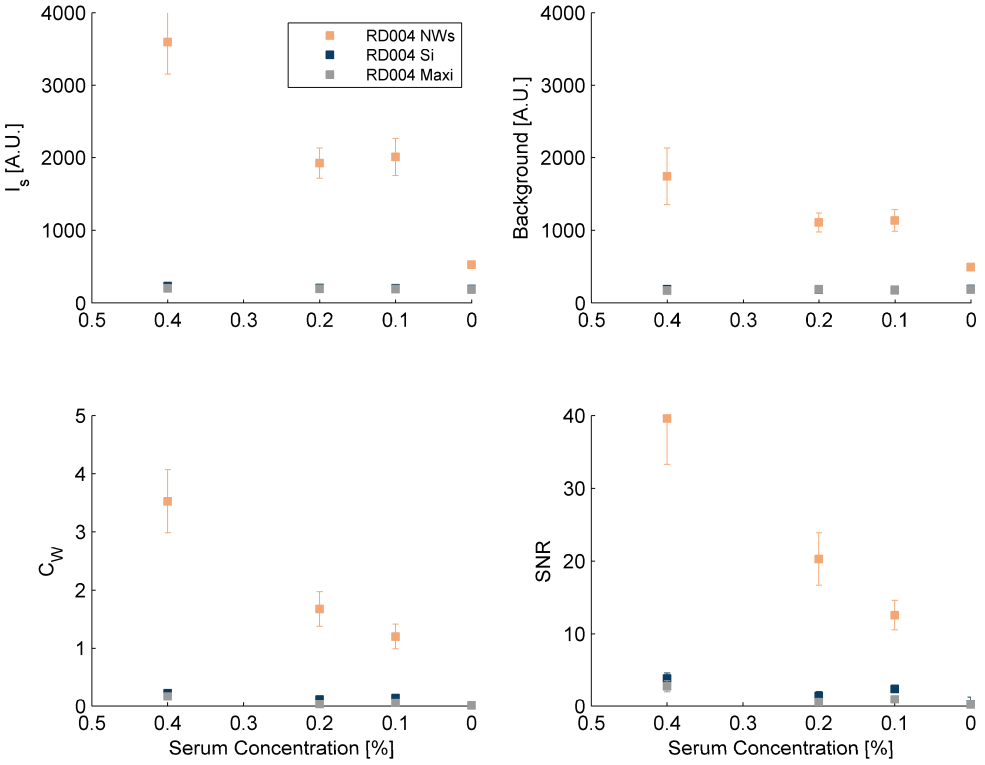
RD004 NWs
RD004 Si
RD004 Maxi
I_s [A.U.]
Background [A.U.]
C_W
SNR
Serum Concentration [%]

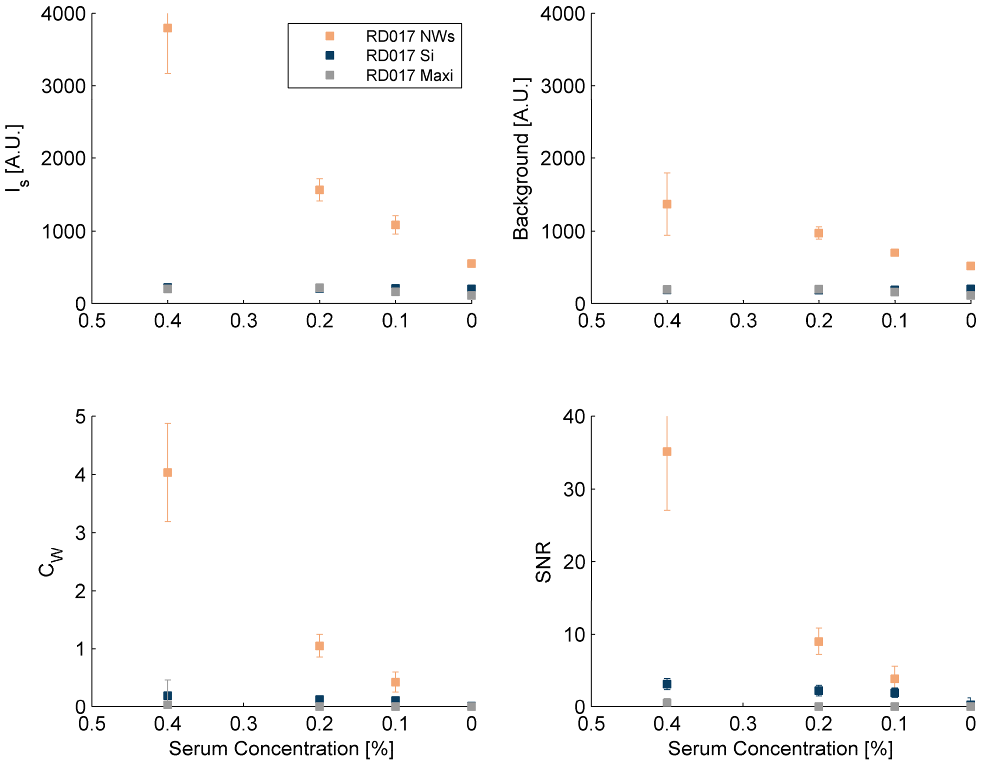

Figure S1: Data for all tested scFvs.

2. Comparison graph in logarithmic scale

Extracted values of signal intensity Is, Weber contrast CW and signal to noise ration SNR for the various scFvs on the three substrates tested with 0.4% serum concentration (as shown in Fig.4 of the main text) in logarithmic scale to better compare flat silicon wafers and MaxiSorp plastic slides. Overall silicon and MaxiSorp samples performed similarly.

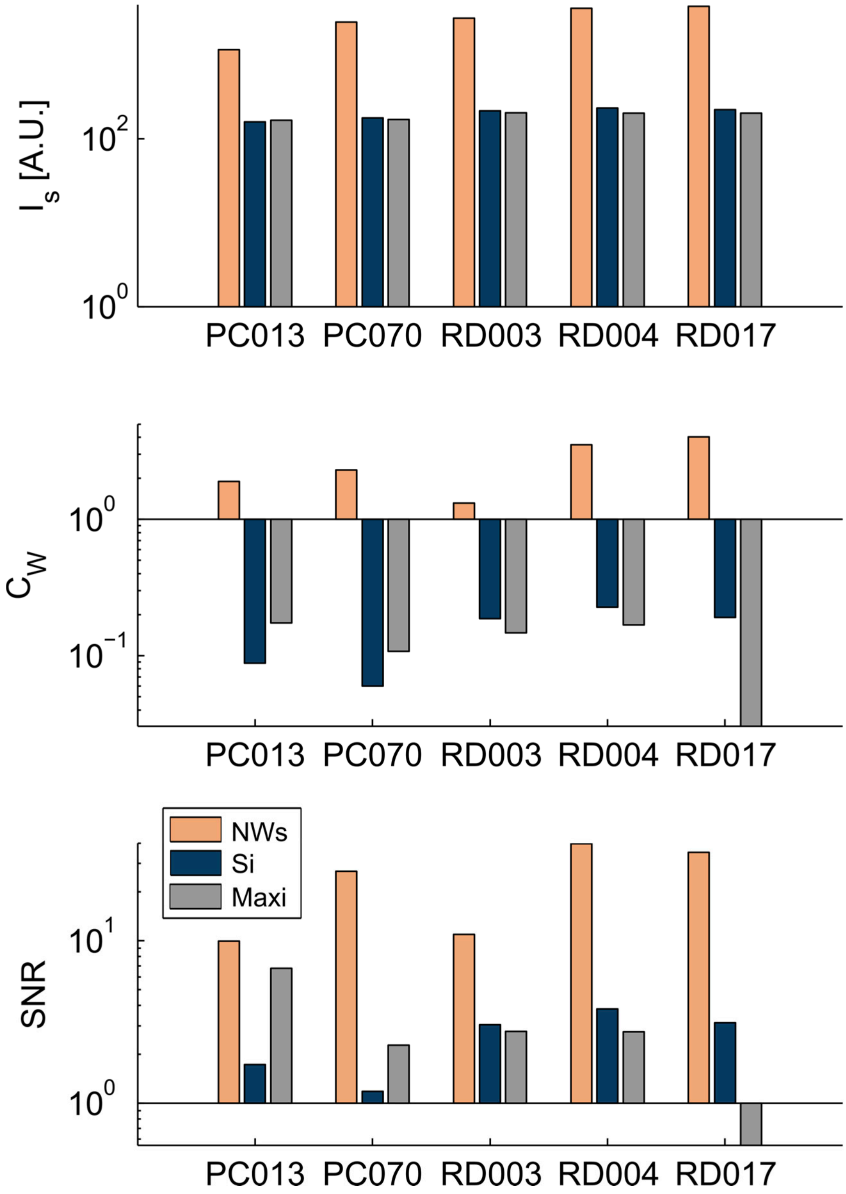

Figure S2: Figure 4a plotted in logarithmic scale.
